# Posttranslational Modifications of Proopiomelanocortin in Vertebrates and Their Biological Significance

**DOI:** 10.3389/fendo.2013.00143

**Published:** 2013-10-17

**Authors:** Akiyoshi Takahashi, Kanta Mizusawa

**Affiliations:** ^1^School of Marine Biosciences, Kitasato University, Sagamihara, Kanagawa, Japan

**Keywords:** acetylation, pigment-dispersing activity, pars distalis, pars intermedia, pituitary, proopiomelanocortin, melanocyte-stimulating hormone, melanocortin receptor

## Abstract

Proopiomelanocortin (POMC) is the precursor of several peptide hormones generated in the pituitary gland. After biosynthesis, POMC undergoes several posttranslational modifications, including proteolytic cleavage, acetylation, amidation, phosphorylation, glycosylation, and disulfide linkage formation, which generate mature POMC-derived peptides. Therefore, POMC is a useful model for the investigation of posttranslational modifications. These processes have been extensively investigated in mammals, primarily in rodents. In addition, over the last decade, much information has been obtained about the posttranslational processing of POMC in non-mammalian animals such as fish, amphibians, reptiles, and birds through sequencing and peptide identification by mass spectrometry. One POMC modification, acetylation, is known to modulate the biological activities of POMC-derived α-melanocyte-stimulating hormone (α-MSH) having an acetyl group at N-terminal through potentiation or inhibition. This bidirectional regulation depends on its intrinsic roles in the tissue or cell; for example, α-MSH, as well as desacetyl (Des-Ac)-α-MSH, stimulates pigment dispersion in the xanthophores of a flounder. In contrast, α-MSH does not stimulate pigment dispersion in the melanophores of the same species, whereas Des-Ac-α-MSH does. Regulation of pigment-dispersing activities may be associated with the subtle balance in the expression of receptor genes. In this review, we consider the posttranslational modifications of POMC in vertebrates from an evolutionary aspect, with a focus on the relationship between acetylation and the biological activities of α-MSH as an important consequence of posttranslational modification.

## Introduction

Proopiomelanocortin (POMC) is a precursor protein of multiple peptide hormones such as adrenocorticotropic hormone (ACTH), melanocyte-stimulating hormone (MSH), endorphin (END), etc. (1). The major tissue that biosynthesizes POMC is the pituitary gland, where POMC is produced in the corticotrophs of the pars distalis (PD) and in the melanotrophs of the pars intermedia (PI) (2, 3). In these cells, POMC is differentially cleaved through tissue-specific proteolysis to generate functional peptides. In corticotrophs, relatively larger peptides such as ACTH are the final products, whereas in melanotrophs, relatively smaller peptides such as α-MSH are generated. In addition to cleavage, POMC and POMC-derived peptides undergo several posttranslational modifications such as acetylation, amidation, phosphorylation, glycosylation, and disulfide linkage formation (4). Therefore, the POMC system is undoubtedly multifunctional, i.e., in addition to the generation of several peptides, various modifications could diversify the biological functions of POMC-derived peptides.

Adrenocorticotropic hormone and MSH are collectively called melanocortin (MC). Their receptor is called the MC receptor (MCR), for which five subtypes (MC1R to MC5R) have thus far been identified (5). The receptors for END are opioid receptors (6, 7). Both receptors are G protein-coupled receptors (GPCRs) with seven transmembrane domains. MCRs are widely distributed throughout animal bodies (8, 9) indicating that POMC-derived peptides have a variety of biological functions. Moreover, posttranslational modification sometimes alters either the binding affinity between the hormonal peptides and their receptors, or the downstream intracellular signal transduction (10). It is thought that the complex POMC network is made up of a variety of peptides with additional modifications and receptor subtypes distributed in many different tissues and organs. Therefore, POMC could be a useful model for investigating posttranslational modifications in endocrine systems.

Posttranslational processing of POMC in mammals is well understood (2, 3). Mammalian POMC is composed of three major segments, *N*-POMC, ACTH, and β-LPH. These segments are divided by the dibasic amino acid residues Arg and Lys, which act as cleavage signals, and contain one MSH sequence whose common sequence is His-Phe-Arg-Trp. The END sequence is always located at the C-terminal end of the β-LPH segment. Therefore, mammalian POMC is described as the 3MSH/1END type. We have investigated the molecular cloning of POMC in non-mammalian species such as birds (11), reptiles (12), and fish, including teleosts, cartilaginous fish, lobe-finned fish, and agnathans (13). Based on the results, we showed the variation in the molecular organization of POMC; the POMC structures are not always the 3MSH/1END type. Moreover, we found that different POMCs are generated in the PD and PI of the most primitive vertebrates, the lampreys (14), whereas identical POMCs are generated in these lobes in other vertebrates. In addition, we also identified POMC-derived peptides from the pituitary glands in non-mammalian vertebrates.

Herein, we compare the posttranslational modifications of POMC in the corticotrophs and melanotrophs in vertebrates such as birds, reptiles, and fish, which are largely based on the results of our investigations. The results for mammalian and amphibian POMCs will also be summarized. Moreover, we also propose a heteromer hypothesis that would explain the interesting activities of α-MSH in relation to its posttranslational modifications, namely the presence or absence of acetyl groups at the N-terminus.

## Posttranslational Processing of POMC

### Mammals

Mammalian POMC is the 3MSH/1END type (1). Posttranslational processing of POMC in the cells of AtT 20/D16v (mouse pituitary epithelial-like tumor cell line) in addition to the PD and PI cells of the pituitary gland has been extensively investigated in mammals such as rodents, including rat and mouse, artiodactyls, including ox and sheep, and humans by peptide isolation/purification and amino acid sequence analysis or by biosynthetic labeling, immunoprecipitation, and sequence analysis (2–4, 15, 16). The results showed that the products from POMC in the PD and PI vary depending on the presence of prohormone convertase 1 and 2 (PC1 and PC2). In the PD, where PC1 is present, pro-γ-MSH, joining peptide (JP), ACTH, and β-LPH are generated; however, in the PI, where PC1 and PC2 are present, pro-γ-MSH is further cleaved to adrenal mitogenic hormone (AMH) and γ-MSH, ACTH is cleaved to generate α-MSH and corticotropin-like intermediate lobe peptide (CLIP), and β-LPH is cleaved to generate *N*-β-LPH, β-MSH, and β-END. α-MSH is produced by way of an intermediate (ACTH_1–17_), and then mature α-MSH is generated after further processing, including removal of C-terminal residues by carboxypeptidase E, formation of a C-terminal amide by peptidyl α-amidating mono-oxygenase, and N-acetylation by POMC *N*-acetyltransferase. Some amount of β-END also undergoes N-terminal acetylation. As adult human pituitary glands lack PI and are only composed of anterior lobes containing the PD and pars tuberalis (17), POMC is predominantly processed into pro-γ-MSH, JP, ACTH, and β-LPH.

### Birds

**Figure 1:**
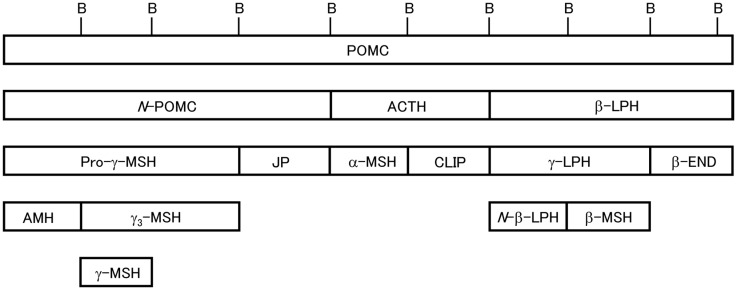
**Schematic of 3MSH/1END type POMC [modified from Ref. (24)]**. Relative positions of the peptide segments are shown. Peptide terminology modified from Eberle (4). B, dibasic sequences that partition the peptide segments. In teleost POMC, *N*-POMC is not divided into subsegments due to the absence of a γ-MSH segment and pairs of basic amino acids.

Similar to human pituitary glands, adult avian pituitary glands are composed of only the PD (18). The ostrich (*Struthio camelus*) is a non-flying bird from which several POMC-derived peptides have been isolated, including ACTH (19), β-LPH (20), β-END (21), γ-LPH (22), and pro-γ-MSH(23), which lacks the C-terminal segment of γ_3_-MSH, and thus is shorter than the pro-γ-MSH as shown in Figure 1. The occurrence of ACTH, γ-LPH, and β-END was further confirmed in a single frozen ostrich pituitary slice through matrix-assisted laser desorption/ionization time-of flight mass spectrometry (MALDI-TOF MS) (11). Based on the results of peptide identification, we cloned the POMC cDNA from ostrich pituitary and determined its sequence. Sequence comparison of these isolated peptides with the POMC cDNA sequence suggests that all the dibasic sequences are cleaved to produce the peptides. Therefore, the major products in ostrich corticotrophs are pro-γ-MSH, ACTH, and β-LPH, although a substantial amount of β-LPH is further cleaved into γ-LPH and β-END. The generation of a substantial amount of β-END in the ostrich pituitary gland is different from what was observed in the human pituitary, in which β-LPH is a predominant form (15).

### Reptiles

**Figure 2:**
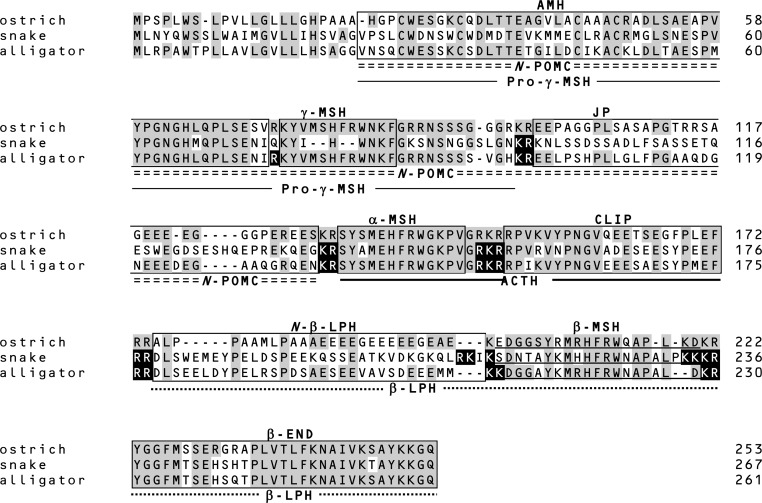
**Amino acid sequences of prePOMC from birds and reptiles**. Amino acid residues in reptile POMC that are identical to those in ostrich POMC are shaded. Filled amino acid residues indicate the processing sites of snake and alligator POMCs determined based on the peptides identified by mass spectrometry and peptide analysis (11, 12). Positions of the amino acid are indicated on the right side.

Similar to other tetrapods, snake and alligator POMCs contain α-MSH, β-MSH, γ-MSH, and β-END (12). These data together with those for gecko (25) and turtle POMC (26) indicate that reptile POMCs are consistently the 3MSH/1END type. Interestingly, the γ-MSH segment in snake POMC has a mutation in the essential His-Phe-Arg-Trp sequence, and the Phe and Arg residues are deleted (Figure 2). It is conceivable that an ancestor of snake γ-MSH had weak functional constraints and lacked biological significance during evolution. In contrast, analyses of whole snake and alligator pituitary glands by MALDI-TOF MS revealed several peptides, such as desacetyl (Des-Ac)-α-MSH, β-MSH, β-END, etc., are generated by posttranslational processing as predicted by the locations of the dibasic sequence processing sites. These results revealed interesting features of the posttranslational processing that generates γ-MSH and β-MSH with reference to the snake POMC as described below.

The γ-MSH segment of snake POMC is characterized by the change in the essential sequence from His-Phe-Arg-Trp to His-Trp (12). Based on this, the term γ-MSH-like sequence was assigned. Moreover, the amino acid residues flanking this segment are Gln-Lys and Lys-Ser at N-terminal and C-terminal sides, respectively. These characteristics suggest that the γ-MSH-like sequence is non-functional and is not liberated from the precursor protein by proteolytic cleavage. This hypothesis was supported by the identification of an *N*-POMC peptide consisting of AMH and γ-MSH-like sequences. The γ-MSH-like sequence seems to be a so-called vestige. The snake POMC is assigned as a 3MSH/1END type on the basis of its overall molecular organization. However, taking its probable lack of a functional γ-MSH into consideration, its direction in evolution is perhaps toward a 2MSH/1END type.

The amino acid sequence of the alligator γ-MSH is identical to the γ-MSH sequences of the leopard gecko, mud turtle, and birds (12). The detection of AMH and JP indicates that alligator POMC is cleaved at Arg^75^ and Arg^89^–Arg^90^ by posttranslational processing; therefore, γ-MSH or γ_3_-MSH must also be liberated. However, these peptides were not detected. Non-detection of γ_3_-MSH suggests that a carbohydrate side chain is probably linked to the C-terminal region of γ_3_-MSH via an N-glycosylation site at alligator prePOMC_91–93_. In contrast, γ-MSH seems not to be liberated from POMC, or in other words, Arg^89^–Arg^90^ are not functional processing signals in alligator POMC.

Despite the consistent presence of the β-MSH sequence in all vertebrate POMCs, β-MSH is not always liberated. In ostriches, β-LPH and γ-LPH peptides, both containing β-MSH, have been detected, whereas the β-MSH peptide has not been detected (11, 27). This is probably caused by the presence of just a single basic residue on the N-terminal side of β-MSH, which constitutes an incomplete processing site. A similar mutation is also observed in rodents (28, 29). In snakes, the processing signal on the N-terminal side of β-MSH is Lys^213^-Ser^214^, while Lys–Lys is observed in other reptiles, whereas its C-terminal side is flanked by four basic residues, Lys^233^-Lys^234^-Lys^235^-Arg^236^ (Figure 2). The detection of β-MSH originating from snake prePOMC_214–232_ indicates that the single N-terminal basic residue and C-terminal four-basic-residue sequence function as cleavage sites. The sequence Arg^210^-Lys^211^-Ile^212^-Lys^213^ on the N-terminal side of the snake β-MSH is similar to a consensus sequence for a monobasic processing site, in which Arg^210^ is especially important (30). Perhaps a synchronous mutation, a changing Lys to Ser at position 214 and substituting Arg for a non-basic residue at position 210, contributes to the liberation of β-MSH in snakes.

### Amphibians

The processing of POMC in African clawed frog (*Xenopus laevis*) has been well investigated. Molecular cloning studies have shown the presence of two forms of 3MSH/1END type POMC (31). POMC-derived peptides have been detected in isolated melanotropic cells from the pituitary NIL consisting of PI and pars nervosa (32–34). The generation of almost all the peptides could be predicted by the presence of mono and dibasic amino acid residues. Further, acetylated forms, such as α-MSH and *N*-Ac-β-END, have also been detected. Therefore, it is probable that *Xenopus* melanotropic cells possess functions similar to those observed in the PI of mammals and reptiles based on the similar posttranslational modifications of their respective POMC-derived peptides

### Fish

#### Lobe-finned fish

The lobe-finned fish include lungfish and coelacanth, and are considered to be the basal members of the lineage that led to the tetrapods (35–37). We demonstrated, for the first time in lobe-finned fish, that African lungfish POMC is the 3MSH/1END type by molecular cloning studies (38). An outline of the posttranslational processing of lungfish POMC has yet to be depicted except for α-MSH. α-MSH was shown to possess an amino acid sequence (based on its cDNA sequence) that is identical to that observed in mammals. Prior to our molecular studies, African lungfish α-MSH was characterized by high-performance liquid chromatography (HPLC) and radioimmunological detection (39). In this experiment, HPLC analysis of African lungfish pituitary extracts showed that the immunological peaks co-eluted with synthetic Des-Ac-α-MSH, α-MSH, and diacetyl (Di-Ac)-α-MSH. This indicated that at least these α-MSH-related peptides are processed and modified in lungfish, probably in the PI, as they are in tetrapod species. Similar experiments have been performed for the Australian lungfish *Neoceratodus forsteri* (40, 41).

No POMC sequence is available for the coelacanth *Latimeria chalumnae*, the other representative lobe-finned fish. However, it is possible to infer a portion of the processing system using the peptide information we obtained. We identified α-MSH, Des-Ac-α-MSH, β-MSH, CLIP, pro-γ-MSH, and *N*-Ac-β-END_1–30_ in an extract from the rostral PD of the pituitary by HPLC, amino acid sequence analysis, and mass spectrometry (42). The occurrence of three different MSHs and one β-END indicates that the structural organization of coelacanth POMC is identical to that of lungfish and tetrapods (3MSH/1END type).

Among these peptides, α-MSH, Des-Ac-α-MSH, and *N*-Ac-β-END_1–30_ are modified by acetylation at the N-terminus or by amidation at the C-terminus. The posttranslational processing of POMC includes cleavage of the precursor into several peptide hormones, modifications of N- and C-terminal residues, and the addition of a carbohydrate moiety (2, 3). Identification of the modified peptides indicated that the coelacanth POMC-related peptides are not produced by autolysis during-transportation or storage after capture of the specimen. Although the major POMC products in the anterior lobe of the pituitary in mammals are *N*-POMC, ACTH, β-LPH, and β-END, small amounts of so-called PI peptides have also been detected in ox, pig, and rat, and significant amounts of PI peptides have been detected in sheep pituitary anterior lobe (2). Therefore, the properties of the coelacanth pituitary rostral PD are similar to those of the anterior lobe of the sheep pituitary.

#### Ray-finned fish

The ray-finned fish include the chondrosteans and neopterygians. Chondrosteans include bichirs and sturgeons, and the neopterygians are divided into teleosts and another group consisting of gars and bowfin (43). POMC in all ray-finned fish, except for teleosts, is the 3MSH/1END type. Indeed, the structures of POMCs from bichir, sturgeon, and gar are similar to those of the lobe-finned fish and tetrapods. However, teleost POMC has distinct features due to the lack γ-MSH in *N*-POMC; therefore, teleost POMC is the 2MSH/1END type [see a review of Ref. (13)]. Based on the POMC-derived peptides identified in several ray-finned fish, including tuna (*Thunnus obesus*), carp (*Cyprinus carpio*), and flounder (*Verasper moseri*), we can infer the posttranslational processing system in teleosts*.*

Initially, extracts from whole pituitary consisting of both PD and NIL were used to identify POMC-derived peptides. These extracts were separated by chromatography, and then the mass value of each peptide was measured by mass spectrometry, and finally the mass value was assigned to an amino acid sequence deduced from the POMC cDNA sequence. Using these methods for carp (44), tuna (45), and flounder POMC (46), most of the peptides predicted from the location of processing signals in the cDNA sequences were identified. However, no PD-specific peptides, such as ACTH and β-END, were detected. These extracts were mixtures of PD and NIL. Morphologically, it is evident that there are relatively few POMC-producing cells in the PD (corticotrophs) compared to the number in the PI (melanotrophs) (47–49). Therefore, it is reasonable to suppose that a lower amount of PD-specific POMC-derived peptides than PI-specific peptides are present in the whole-pituitary extracts, and that analyses of whole pituitaries may better represent the peptide profiles in the PI.

To identify PD- and PI-specific POMC-derived peptides, pituitaries were taken from relatively large individual flounder, and the pituitaries were divided into the PD and NIL. The tissues were used for direct profiling of the pituitary slice by MALDI-TOF MS (50). A mass value identical to the estimated value from the cDNA sequence was detected in the PD, indicating that ACTH is generated in the corticotrophs of the flounder pituitary. Moreover, Des-Ac-α-MSH and CLIP were also identified in the PD. These findings indicate that in flounder, a substantial amount of ACTH is further processed to generate these two peptides in corticotrophs. Although β-END was not detected in the PD, the presence of β-MSH indicates that the dibasic sequence Lys-Arg between β-MSH and β-END is part of the processing signal for cleavage in the flounder pituitary PD. Cleavage at this signal sequence should also generate β-END, which is located at the C-terminal end of POMC.

In contrast, ACTH-derived peptides, such as Des-Ac-α-MSH, α-MSH, and CLIP, but not ACTH itself, were identified in the flounder PI. In melanophores, most of the ACTH seems to be cleaved into Des-Ac-α-MSH and CLIP, then an acetyl group is added to the N-terminus of Des-Ac-α-MSH to form α-MSH.

#### Cartilaginous fish

Cartilaginous fish (chondrichthians) are composed of elasmobranchs, including sharks and rays, and holocephalans, including ratfish (43). The amino acid sequence of POMC from cartilaginous fish was first reported for the elasmobranch dogfish (*Squalus acanthias*), which was determined by molecular cloning studies (51). Subsequently, sequence information has been obtained in rays (52), ratfish (53), and other sharks (54, 55). The presence of δ-MSH in the *N*-β-LPH segment is a distinctive feature of cartilaginous fish POMC. Therefore, cartilaginous fish POMCs are the 4MSH/1END type.

The occurrence of the δ-MSH peptide as well as other types of MSH was initially shown through peptide chemical analyses using whole pituitaries (56). Tissue-specific cleavage of POMC was demonstrated in the banded houndshark (*Triakis scyllium*) using PD and NIL extracts (55). In the *Triakis* POMC, predicted segments including δ-MSH were flanked by dibasic sequences, as has been observed for POMCs from other fish and animals. Mass spectrometry was performed on PD (including most parts of the rostral and proximal PD) and NIL extracts to detect mass values corresponding to POMC-derived peptides. Consequently, ACTH, β-END, and JP were detected in the PD extract, whereas MSHs, such as α-, β-, γ-, and δ-MSH, truncated β-ENDs, such as β-END_1–30_, and other POMC-derived peptides, such as JP and CLIP, were identified in the NIL extract. It is apparent that larger peptides than those found in the NIL are generated in the PD.

Based on the *Triakis* POMC sequence, pro-γ-MSH and γ-LPH should also be liberated in the pituitary PD, although these were not detected (55). Detecting these relatively large peptides might be more difficult than detecting the other small POMC-derived peptides. In contrast, in the NIL, the only two peptides whose peaks could not be identified by mass spectrometry were the C-terminal peptides of γ_3_-MSH and *N*-β-LPH. The former peptide probably contains a carbohydrate side chain, as suggested by the presence of an N-glycosylation site at POMC_93–95_. The latter peptide is highly acidic. These physicochemical characteristics might have interfered with the mass spectrometric detection of these peptides.

#### Agnathans

Although an identical POMC gene is expressed in the both the PD and PI of the pituitary gland in jawed vertebrates, two distinct POMC genes are expressed in jawless fish, the most primitive vertebrates (14, 57). In lampreys, an mRNA encoding proopiocortin (POC), the precursor of ACTH and β-END, is synthesized in the corticotrophs of the PD, while an mRNA encoding proopiomelanotropin (POM), the precursor of MSH and the other β-END, is synthesized in the melanotrophs of the PI (14, 57, 58). POC is a 1MSH/1END type and POM is a 2MSH/1END type.

**Figure 3:**
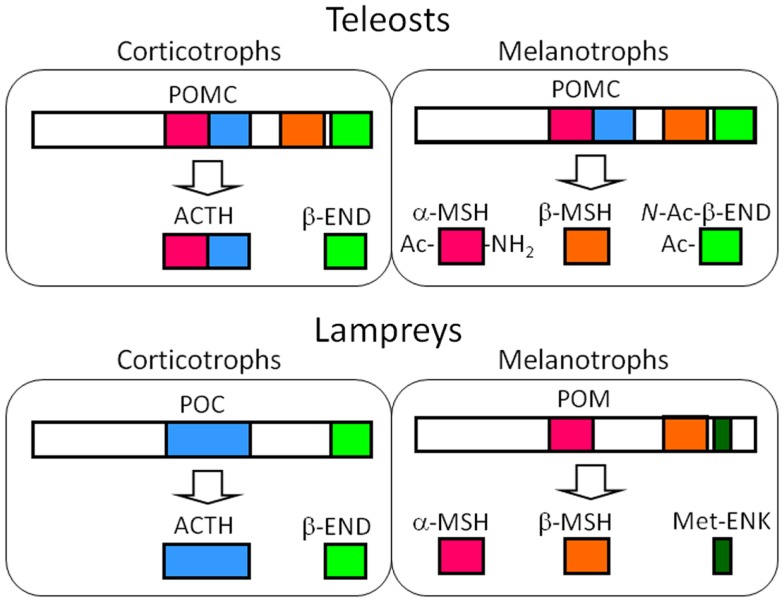
**Major POMC-derived peptides in the corticotrophs of the PD and the melanotrophs of the PI in teleost and lamprey pituitary glands based on the results from the barfin flounder (*Verasper moseri*) (14, 46, 50) and sea lamprey (*Petromyzon marinus*) (14, 57–59)**. In the original paper on the sea lamprey POMC, α-MSH, and β-MSH are termed MSH-B and MSH-A, respectively (59). The major products in these cells are common between teleosts and lampreys – ACTH and END in the melanotrophs, and MSHs and opioid peptides, i.e., *N*-Ac-β-END or Met-ENK. The differences between teleosts and lampreys lie in the genes expressed; identical POMCs are generated in the corticotrophs and melanotrophs of teleosts, as is the case for tetrapods. In contrast, different precursors are generated in lampreys.

Initially, ACTH and the two types of MSH were isolated from whole-pituitary extract of lamprey (59). Subsequently, mass spectrometric analyses demonstrated the tissue sources for each peptide, including β-END (60). In brief, the peptides that originated from POC, such as ACTH and β-END, were shown to be produced in the PD, and those that were from POM, such as MSH-A, MSH-B, and β-END_8–35_ lacking the N-terminal Met-enkephalin (Met-ENK) region, were shown to be produced in the PI. The occurrence of β-END_8–35_ indicates that the dibasic Arg-Lys sequence at β-END_6,7_ functions as the processing signal for cleavage in lamprey. Cleavage at β-END_6,7_ should also generate β-END_1–5_, namely Met-ENK. The occurrence of Met-ENK in the PI of the lamprey pituitary was first proposed by Dores and McDonald (61) who fractionated a PI extract by HPLC. In this study, immunoreactive Met-ENK had a retention time that was identical to synthetic Met-ENK. Taken together, it is indicated that the major products generated through posttranslational processing are ACTH and β-END in the PD, and MSHs and Met-ENK in the PI (Figure 3).

Despite the expression of different genes, the roles of the POMC-producing cells in the each lobe of the lamprey are basically the same as those observed in jawed vertebrates; ACTH is a PD peptide and MSH is a PI peptide (14, 57, 58, 60). However, the generation of Met-ENK is an obvious difference between lampreys and jawed vertebrates. It is conceivable that the dibasic sequence located between Met-ENK and the rest of the β-END sequence undergo enzymatic cleavage. Therefore, the melanotrophs of lamprey pituitaries are a source of MSH and Met-ENK, which is different from their roles in jawed vertebrates. Based on the final products, POM should be the precursor of MSHs and Met-ENK, but not of β-END.

## Acetylation: Evolutionary Implications

Acetylation of the N-termini of Des-Ac-α-MSH and β-END, phosphorylation of ACTH, and glycosylation of *N*-POMC are the representative posttranslational modifications of POMC or POMC-derived peptides (2–4). Acetylation is known to affect the biological function of POMC-derived peptides (10). Between the two lobes in which POMC is generated, acetylation frequently occur in the PI.

Desacetyl-α-MSH is an N-terminal fragment of ACTH with an amide at the C-terminus that is identical to ACTH_1-13_-NH_2_ (4). α-MSH has one acetyl group at the N-position of the N-terminal Ser residue, whereas Di-Ac-α-MSH has an additional acetyl group at the O-position of the N-terminal residue. In *Xenopus* α-MSH, the N-terminal residue, Ala, is the residue to which the acetyl group is added (34). In the case of β-END, the N-terminal Tyr residue is acetylated at the N-position (2, 3). The acetylated peptide is termed *N*-Ac-β-END.

Proopiomelanocortin-derived peptides containing acetyl groups, such as α-MSH, Di-Ac-α-MSH, and *N*-Ac-β-END, have been detected in fish, including teleosts (44, 45, 50, 62–65) and lobe-finned fish (39, 40, 42), and tetrapods, including amphibians (34), reptiles (12), and mammals, such as rodents and artiodactyls (2, 3). No acetylated forms of POMC-derived peptides have been detected in agnathans (59), cartilaginous fish (55), and birds (11). Considering the phylogenetic relationships of vertebrates, the ability of acetylation or the presence of a functional *N*-acetyltransferase in the melanotrophs of the PI may have been established in a common ancestor of fish and tetrapods. According to this assumption, cartilaginous fish may have secondarily lacked these acetylation systems. In contrast, the presence of a few acetylated POMC-derived peptides in the pituitary glands in birds and human corresponds to the lack of the PI in the pituitary gland.

## Acetylation in Relation to Biological Activities

The presence or absence of acetyl groups modifies the biological activities of POMC-derived peptides (10). α-MSH-related peptides, such as Des-Ac-α-MSH, α-MSH, and Di-Ac-α-MSH, differentially affect melanogenesis, pigment migration in chromatophores, neural development and regeneration, melanocortinergic regulation of food intake and other behaviors, corticosteroid genesis, lipolysis, cell proliferation in bones, and lactotrope-recruiting activity. These differences may result from an altered interaction between the ligand and receptor. For instance, α-MSH showed significantly higher activities than Des-Ac-α-MSH, and Di-Ac-α-MSH is as potent as α-MSH (66–68). In contrast, *N*-Ac-β-END has no opiate activity because acetylation of β-END significantly decreased its interaction with opioid receptors (69). In this chapter, we summarize the new concept of ligand-receptor interaction based on the results of studies on pigment migration in flounder (*Verasper moseri*).

**Figure 4:**
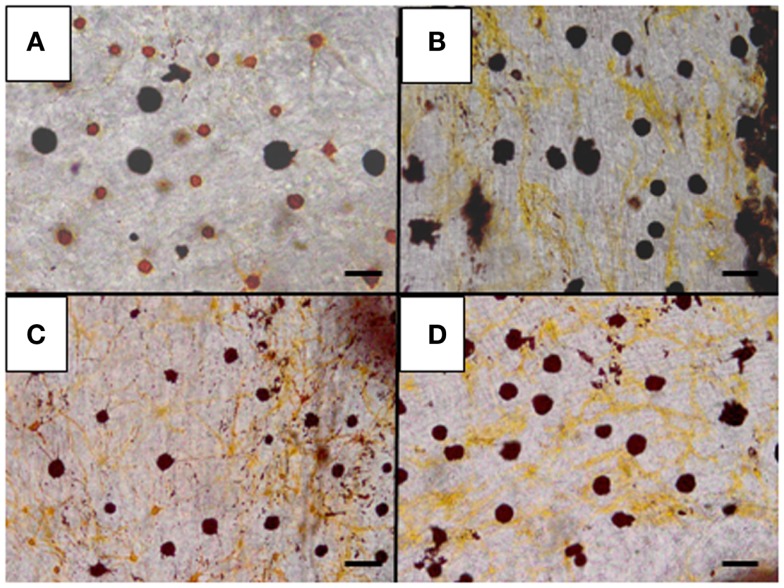
**Effects of α-MSH on pigment dispersion in chromatophores in the skin of barfin flounder**. A control skin specimen was incubated in medium containing no α-MSH **(A)**. Skin specimens were treated with 10 nM **(B)**, 100 nM **(C)**, and 1 μM α-MSH **(D)** at 16°C for 1 h. Pigments in melanophores did not migrate, whereas those in xanthophores diffused. See Kobayashi et al. (71) for details. The bar equals 100 μm.

In *Verasper* skins two types of chromatophores – melanophores and xanthophores – are predominantly observed. α-MSH and Des-Ac-α-MSH exhibit pigment-dispersing activities in both melanophores and xanthophores. However, N-terminal acetylation differentially modulates the pigment-dispersing activities of these cells (70). Surprisingly, acetylation significantly decreased the pigment-dispersing activity of α-MSH in melanophores, while it stimulated the pigment-dispersing activity in xanthophores. Similar results were also obtained for another flounder species, *Paralichthys olivaceus* (Figure 4) (71). At first, it was thought that α-MSH has a low affinity for the MCR expressed in melanophores (70).

There are at least five subtypes of MCR (MC1R–MC5R) in fish, as in tetrapods (72–75), while *Fugu* lack MC3R (74, 76) and possess only four MCRs. We also identified the four subtypes of MCRs in *Verasper* (77–79). Among the subtypes, *Mc5r* transcript was detected in xanthophores, whereas *mc1r* and *mc5r* transcripts were detected in melanophores (78). It was reasonable to conclude that both Des-Ac-α-MSH and α-MSH stimulated pigment dispersion via MC5R on xanthophores. However, roles of two MCRs in the melanophores is still unclear.

**Figure 5:**
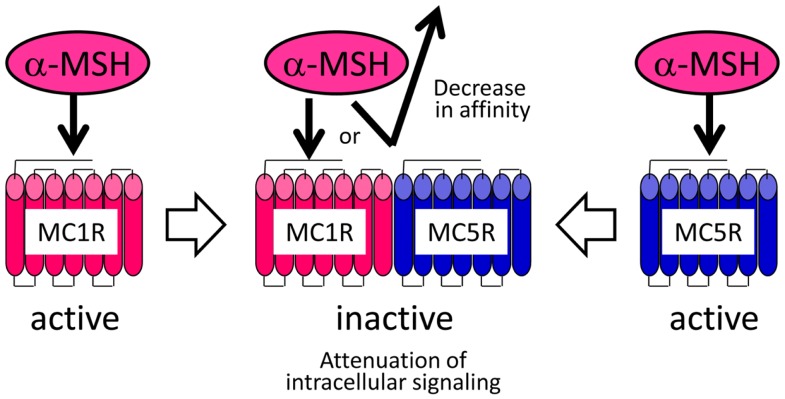
**Heteromer hypothesis explaining the negligible activities of α-MSH in flounder melanophores**.

Although the pharmacological properties of the *Verasper* MCRs have not yet been determined, they may have properties similar to those of the sea bass (*Dicentrarchus labrax*) MCRs because *Verasper* MC1R and MC5R share approximately 90% amino acid sequence identity with sea bass MC1R and MC5R. Interestingly, pharmacological studies of sea bass MC1R and MC5R have revealed that α-MSH has a much higher efficacy than Des-Ac-α-MSH in stimulating cAMP production in human embryonic kidney (HEK) 293 cells, which stably expressing either *mc1r* or *mc5r* (66, 68). If the properties of *Verasper* MCRs in HEK293 cells are similar to those of the sea bass MCRs, the negligible effects of α-MSH in melanophores is inconsistent with the presence of the MC1R and MC5R because these receptors can mediate the α-MSH signal. To resolve these discrepancies, we postulated the formation of a heteromer consisting of MC1R and MC5R based on increasing evidence which suggests that heterodimerization of GPCRs results in functional consequence that are different from the well-established functional characteristics of monomeric receptors (80–82). The keys to this assumption are that this putative heteromer mediates signals of Des-Ac-α-MSH, but not those of α-MSH, which would differ from the properties of each monomeric MCR (Figure 5). α-MSH seems to have a low binding affinity for the putative heteromer consisting of MC1R and MC5R in melanophores, or this peptide cannot stimulate intracellular signal transduction through these heteromers even though interaction would occur between the peptide and receptor. Our findings seem to suggest the presence of naturally occurring heteromeric GPCRs.

## Conclusion

Examination of the POMC-derived peptides in a variety of vertebrates demonstrated that the posttranslational modification (or final products) of POMC in the corticotrophs is different from those in the melanotrophs. Relatively larger peptides are generated in the corticotrophs, while smaller peptides are generated in the melanotrophs. Moreover, some peptides in the melanotrophs undergo acetylation. These can be considered common processes that generate tissue-specific POMC-derived peptides in vertebrates; however, deviations in the proteolytic cleavage and acetylation are sometimes observed. Nevertheless, we can summarize that the common function of corticotrophs throughout the vertebrates is the production of ACTH and the opioid peptide β-END, and that the common function of melanotrophs is the production of MSH and the opioid peptides β-END and Met-ENK. This functional differentiation may have been established in the evolutionary antecedent of lampreys. α-MSH and β-END are frequently acetylated, and the presence or absence of an acetyl group modifies the biological activities in many vertebrate classes. The lack of acetylated peptides in the PI of lamprey and cartilaginous fish pituitaries suggests that acetylation is not a commonly occurring modification in vertebrates. However, the presence of an acetylation system in tetrapods and teleosts suggests the establishment of this system in their common ancestor. Acetylation may be associated with the interaction of the receptor molecules to regulate the roles of POMC-derived peptides. In this context, lampreys and cartilaginous fish lacking acetylation systems may have different regulating systems for the peptide generation in the melanotrophs from teleosts and tetrapods.

## Conflict of Interest Statement

The authors declare that the research was conducted in the absence of any commercial or financial relationships that could be construed as a potential conflict of interest.
